# Recognition of speech in noise and relations with suppression of otoacoustic emissions and the acoustic reflex

**DOI:** 10.1590/S1808-86942011000100019

**Published:** 2015-10-19

**Authors:** Larissa Lautenschlager, Tania Tochetto, Maristela Julio Costa

**Affiliations:** 1 Santa Maria Federal University (UFSM-RS). Speech therapist; 2 Speech therapist, UFSM-RS graduate. UNIFESP. Associate professor, Speech Therapy Department, UFSM-RS; 3 Speech therapist, UFSM-RS graduate. UNIFESP. Associate professor, Speech Therapy Department, UFSM-RS

**Keywords:** adult, efferent pathways, reflex acoustic, hearing tests, acoustic

## Abstract

Subjects presenting difficulties in understanding speech with competing sounds may have absence of otoacoustic emission suppression and the acoustic reflex.

**Aim:** To study the performance of the efferent auditory system in normal hearing subjects complaining of difficulties to understand speech in noise.

**Material and methods:** A prospective study comprising 50 normal-hearing subjects aged from 19 to 32 years, reporting difficulties with speech recognition in noise (with complaints - WC) or not (with no complaints - WNC). Distortion product evoked otoacoustic emissions (DPOAEs) were tested at frequencies from 1500 to 6000 Hz.The contralateral acoustic reflex (CAR) was investigated from 500 to 4000 Hz.

**Results:** Groups differed statistically as to the occurrence of CAR in the left ear at 4000 Hz. At 1500 Hz, there was a statistically significant effect - absence of DPOAEs in the WC group in the right ear. In left ears, absence of DPOAE suppression was higher in the WC at 1500 Hz and 2000 Hz.

**Conclusion:** An association between self-reported difficulties in discriminating speech in noise and the absence of contralateral acoustic reflex at 4000 Hz in the left ear was observed; there was also absence of the suppression effect of DPOAEs, especially at middle frequencies in both ears.

## INTRODUCTION

The efferent auditory system may be evaluated with objective non-invasive methods, such as suppression of otoacoustic emissions (OAEs) and the acoustic reflex.^1^

Suppression of OAEs arises when noise is applied contralaterally, ipsilaterally or bilaterally to the examined ear. This technique assesses the activity of the medial olivocochlear efferent system; two likely functions of this system are to facilitate location of sound sources and to help discriminate sound in the presence of competing noise.^2^

Testing OAE suppression may help evaluate and diagnose difficulty to understand speech in noise, where the suppression effect is absent.^3^

Testing the contralateral acoustic reflex also yields important information about the auditory efferent system at the brainstem.^4^

The hypothesis underlining this study was that the auditory efferent system of volunteers could be dysfunctional, evidenced by lack of the suppression effect of OAEs and the AR, based on self-reported complaints of difficulty to understand speech in the presence of competing noise.

Thus, the purpose of this study was to analyze the suppression effect of OAEs and the contralateral acoustic reflex in normal-hearing subjects with and without difficulties in understanding speech in the presence of noise.

## MATERIALS AND METHODS

A cross-sectional descriptive non-experimental quantitative study was made of data gathered at an audiology laboratory of a speech therapy unit in a federal public institution. After approval from the institutional review board of the institution (approval no. 0131.0.243.000-08), data started to be gathered.

Participants agreed with the study objective and method, and signed a free informed consent form (Resolution 196/1996).

The exclusion criteria were: tinnitus, hyperacusis, signs of middle ear involvement, and other auditory conditions except for self-reported difficulty to understand speech in noise.

The sample comprised 50 normal-hearing subjects aged from 19 to 32 years that reported or not difficulty in understanding speech in noise. There were 24 subjects with the complaint (group CQ) and 26 subjects without the complaint (group SQ). There were 21 male and 29 female subjects.

A clinical history was taken to record personal identification data, auditory complaints and the ontological history. Next, the outer ear canal was inspected, after which pure tone audiometry at 250 to 8000 Hz (air conduction) and at 500 to 4000 Hz (bone conduction) was done. The audiometry device was a Fonix model FA-12 type I two-channel audiometer with Telephonics TDH-39P in-ear phones. Subjects were considered normal hearing when pure tone thresholds were from 0 to 25 dB.

An Interacoustics AZ7 middle ear analyzer with a TDH-39 earphone and MX-41 pad, and a 220 Hz at 70 dBHL tone-probe for tympanometry, ISO 389-1991 calibrated, was used for acoustic immittance testing. Only subjects with type A tympanometric curves - indicating normal mobility of the tympanic-ossicular system - were kept in the study.^5^

Subjects without a type A tympanometric curve and a subject that complained of tinnitus were excluded.

The contralateral acoustic reflex was tested at 500, 1000, 2000 and 4000 Hz and was considered as present when evoked from 70 to 100 dB over the auditory threshold.^6^ If absent, the contralateral acoustic reflex was retested for confirmation purposes. The ipsilateral acoustic reflex was not tested because appropriate equipment was not available.

Distortion product otoacoustic emissions (DPOAEs) recordings were made in an acoustic booth with an Interacoustis/Audiotest Clinical Otoread. Two pure tones (F2/F1 =1.22) were applied for DPOAEs (2F1-F2); the intensity of F1 was 65 dBSPL and of F2 was 55 dBSPL. Both ears were tested at 1500, 2000, 3000, 4000, 5000 and 6000 Hz. DPOAEs were considered as present when the signal-to-noise ratio was at least 6 dB.

DPOAEs were recorded in the absence and in the presence of noise in the contralateral ear.

The suppressive acoustic stimulus was white noise applied contralaterallly (generated by a Fonix model FA12 type I two-channel digital audiometer with Telephonics TDH-39P in-ear phones) at 60 dBHL.

The earphone was placed on the contralateral ear (to the DPOAE-tested ear) before testing to avoid handling the probe during the test.

Contralateral calculation of DPOAE suppression was done by subtracting the response level of DPOAEs with contralateral acoustic stimulation from the response level of DPOAEs without contralateral acoustic stimulation.^7^ Negative values indicated suppression of DPOAEs and positive values or zero indicated that DPOAEs were not suppressed.

The suppression effect of DPOAEs was measured according to the following point:

to assess whether subjects had suppression of DPOAEs, it was considered as present when occurring in at least four of six tested frequencies in each ear.
occurrence of DPOAE suppression per frequency in each ear.

The results were stored in a data base written in Microsoft Excel 2007; the privacy of study subjects was preserved and the data was kept confidential.

Fisher's exact test was applied to investigate the level of association between results. The statistical significance level was < 0.05.

## RESULTS

The presence of contralateral acoustic reflexes in the right ear did not differ statistically between groups; but absence of this phenomenon predominated in the CQ groups, mainly at 4000 Hz (Table 1).

**Table 1:** Comparative analysis of groups CQ and SQ for right ear contralateral acoustic reflexes

500Hz	CAR present n %	CAR absent n %	Fisher's exact test
CQ	22 44	2 4	
SQ	26 52	0 0	*p* = 0,2253
1000Hz			
CQ	22 44	2 4	
SQ	26 52	0 0	*p* = 0,2253
2000Hz			
CQ	22 44	2 4	
SQ	26 52	0 0	*p* = 0,2253
4000Hz			
CQ	17 34	7 14	
SQ	24 48	2 4	*p* = 0,0693

Key: CQ = group with a complaint of speech recognition difficulty in the presence of noise; SQ = group without a complaint of speech recognition difficulty in the presence of noise; CAR = contralateral acoustic reflex.

The results of contralateral acoustic reflexes in right ears are close to those of left ears; there were more absences of contralateral acoustic reflexes at 4000 Hz compared to other frequencies.

Groups CQ and SQ differed statistically with regards to the presence of left ear contralateral acoustic reflexes only at 4000 Hz (p=0,0094). At other frequencies, contralateral acoustic reflexes were mostly absent in the CQ group (Table 2).

**Table 2:** Comparative analysis between groups CQ and SQ of contralateral acoustic reflexes in left ears.

500Hz	CAR present n %	CAR absent n %	Fisher's exact test
CQ	22 44	2 4	*p* = 0,2253
SQ	26 52	0 0	
1000Hz			
CQ	23 46	1 2	*p* = 0,4800
SQ	26 52	0 0	
2000Hz			
CQ	22 44	2 4	*p* = 0,2253
SQ	26 52	0 0	
4000Hz			
CQ	16 32	8 16	*p* = 0,0094*
SQ	25 50	1 2	

Key: CQ = group with a complaint of speech recognition difficulty in the presence of noise; SQ = group without a complaint of speech recognition difficulty in the presence of noise; CAR = contralateral acoustic reflex.

DPOAE suppression was absent at 1500 Hz in the right ear in the CQ group, which was statistically significant (p=0,405) (Table 3). DPOAE suppression was predominantly absent at 2000 and 3000 Hz in the CQ group, although this difference was not statistically significant between groups (Table 3).

**Table 3:** Presence or absence of DPOAE suppression in groups CQ and SQ at specific frequencies in right ears.

1500Hz	Suppression present n %	Suppression absent n %	Fisher's exact test
CQ	11 22,4	12 24,5	*p* = 0,0405*
SQ	19 38,8	7 14,3	
2000Hz			
CQ	12 24	12 24	*p* = 0,8384
SQ	17 34	9 18	
3000Hz			
CQ	14 28	10 20	*p*=0,2069
SQ	20 40	6 12	
4000Hz			
CQ	15 30	9 18	*p*=0,7122
SQ	14 28	12 24	
5000Hz			
CQ	9 18	15 30	*p* = 0,8536
SQ	8 16	18 36	
6000Hz			
CQ	10 20	14 28	*p* = 0,4725
SQ	11 22	15 30	

Key: DPOAEs = distortion product otoacoustic emissions CQ = group with a complaint of speech recognition difficulty in the presence of noise; SQ = group without a complaint of speech recognition difficulty in the presence of noise.

Absence of DPOAE suppression in the left ear was more evident at 1500 Hz (p=0.0085) and at 2000 Hz (p=0.0129) in the CQ group (Table 4). The number of subjects with the DPOAE suppression effect was higher in the SQ group.

**Table 4:** Presence or absence of DPOAE suppression in groups CQ and SQ at specific frequencies in left ears.

1500Hz	Suppression present n %	Suppression absent n %	Fisher's exact test
CQ	9 18,4	14 28,5	*p* = 0,0085*
SQ	20 40,8	6 12,3	
2000Hz			
CQ	11 22	13 26	*p* = 0,0129*
SQ	20 40	6 12	
3000Hz			
CQ	9 18,4	14 28,5	*p* 0,0969
SQ	17 34,7	9 18,4	
4000Hz			
CQ	10 20	1428	*p* = 0,5537
SQ	15 30	11 22	
5000Hz			
CQ	7 14	17 34	*p* = 0,7932
SQ	11 22	15 30	
6000Hz			
CQ	8 16	16 32	*p* = 0,5602
SQ	10 20	16 32	

Key: DPOAEs = distortion product otoacoustic emissions; CQ = group with a complaint of speech recognition difficulty in the presence of noise; SQ = group without a complaint of speech recognition difficulty in the presence of noise.

There was no association between the presence of contralateral acoustic reflexes and DPOAE suppression in both ears, in the complete sample (Tables 5 and 6).

**Table 5:** Presence or absence of contralateral acoustic reflexes and DPOAE suppression in right ears of the entire sample

CAR 500Hz	Suppression present n %	Suppression absent n %	Fisher's exact test
Present	26 52	22 44	*p* = 0,4971
Absent	24	00	
CAR 1000Hz			
Present	26 52	22 44	*p* = 0,4971
Absent	24	00	
CAR 2000Hz			
Present	26 52	22 44	*p* = 0,4971
Absent	24	00	
CAR 4000Hz			
Present	24 48	18 36	*p* = 0,7181
Absent	48	48	

Key: CAR = contralateral acoustic reflex; DPOAEs = distortion product otoacoustic emissions.

**Table 6:** Presence or absence of contralateral acoustic reflexes and DPOAE suppression in left ears of the entire sample

CAR 500Hz	Suppression present n %	Suppression absent n %	Fisher's exact test
Present	21 42	27 54	
Absent	00	24	*p*=0,5029
CAR 1000Hz			
Present	21 42	28 56	
Absent	00	1 2	*p* = 1,0000
CAR 2000Hz			
Present	21 42	27 54	
Absent	00	24	*p*=0,5029
CAR 4000Hz			
Present	16 32	25 50	
Absent	5 10	48	*p*=0,4642

Key: CAR = contralateral acoustic reflex; DPOAEs = distortion product otoacoustic emissions.

## DISCUSSION

The relevance of studying OAE suppression and the contralateral acoustic reflex is well-known, since there is a common physiology between their pathways in the auditory nervous system. Both share the same afferent pathway, the auditory nerve. The efferent responses of OAE suppression and the contralateral contralateral acoustic reflex originate in the superior olivary complex and are recorded in the outer ear.^8^ Thus, both procedures yield objective conditions to analyze efferent pathways using different approaches.

As evoking the acoustic reflex pertains to the auditory efferent system, and that one of the functions of this system is to protect the cochlea against loud noise, it may be stated that this mechanisms is more sensitive in subjects with difficulty to understand speech in competing noise.^9^ The result is that speech understanding in competing noise is compromised, as auditory efferent fibers are activated in the presence of loud noise, altering the mechanics of outer hair cells, thereby reducing the quality of the sound message.^10^

The contraction of intratympanic muscles and the ability to discriminate sounds in the presence of noise are regulated by the superior olivary complex;^11^ thus, it is possible that altered acoustic reflexes may compromise speech intelligibility in noisy environments.

Absence of contralateral reflexes in the right ear, mainly at 4000 Hz, tended to reach a statistically significant difference between the CQ and SQ groups (Table 1). Thus, this variable should not be ignored; if the study sample were larger, statistical significant might have been attained because the trend was close to statistical significance (p=0,0693). The number of subjects in the CQ group without contralateral acoustic reflexes was clearly higher than those in the SQ group (Table 1).

There was a statistically significant difference between the CQ and SQ groups with regards to the presence of contralateral acoustic reflexes at 4000 Hz in left ears (p=0,0094). Absence of contralateral acoustic reflexes predominated at other frequencies in the CQ group (Table 2). These results are similar to those in other papers in that when comparing groups with and without altered auditory processing, the mean values require to evoke the contralateral acoustic reflex in study groups were higher in both ears than those in control groups, especially at 4000 Hz.^9^ We did not find any explanation in the literature about why these findings occur mostly at 4000 Hz.

The findings that the study group had less DPOAE suppression at specific frequencies (Tables 3 and 4) and more absence of the acoustic reflex (Tables 1 and 2), especially at 4000 Hz, may be related with the intensity of the stimuli used in both procedures, which may have activated different areas of the auditory efferent system.

Our results show that there was a higher rate of DPOAE suppression in both ears at middle frequencies. There was a statistically significant predominance of absent DPOAE suppression in right ears in the CQ group at 1500 Hz (p=0,405) (Table 3). Findings trended towards a statistically significant difference between groups at 2000 and 3000 Hz; there was a clear predominance of absent DPOAE suppression in the CQ group (Table 3).

Absence of DPOAE suppression was higher in left ears in the CQ group at 1500 Hz (p=0,0085) and at 2000 Hz (p=0,0129) (Table 4). The number of subjects with the suppression effect was higher in the SQ group.

Our findings agree with those in other papers that have reported a higher rate of DPOAE suppression in normal-hearing subjects without auditory complaints at 1 to 2 KHz rather than at 4 to 6 KHz in both ears and at all age groups, but mostly in adults.^12^

Our results are also similar with those of another study showing that the mean suppression values in controls were higher than those in the study group, which suggested a decreased inhibitory effect in the auditory efferent system of subjects with disordered auditory processing.^9^

There was no agreement with regards to the presence of contralateral acoustic reflexes and DPOAE suppression in both ears in the entire sample (Tables 5 and 6). Thus, we suggest that the efferent function assessed by DPOAE suppression testing is related with improved speech understanding in noisy environments, as ambient noise has a similar intensity to that of the test. The intensity of stimuli applied for evoking contralateral acoustic reflexes is higher and potentially harmful to the cochlea if continued for longer time periods. Thus, we may infer that efferent activity assessed by the contralateral acoustic reflex is related with protection of the cochlea against loud sounds.^13^

Further studies of objective methods for assessing subjects complaining of difficulty to understand speech in noisy environments are needed; our results showed that this population had different responses for DPOAE suppression and contralateral acoustic reflexes.

Doubts remain about the auditory efferent system and its possible association with difficulty in detecting stimuli in the presence of competing noise; thus, our results support the need for further studies of these variables to shed light on diseases that may involve absence of the suppression effect and acoustic reflexes.

## CONCLUSION

We found that the activity of the medial olivocochlear system in normal-hearing subjects that self-reported difficulty in understanding speech in the presence of noise should be investigated. This is because we concluded that acoustic reflexes and OAE suppression are absent at specific frequencies in both ears of subjects with the abovementioned complaint.
